# Office Visitors

**Published:** 1906-10

**Authors:** 


### OFFICE VISITORS.

She was well onto sixty years of age, thin, slim, tall, but of good carriage and erect figure. She had bright blue eyes with a merry twinkle in them and quick of speech, but with a softness not often accompanying the faculty of ready repartee. She was an old maid and we wondered why, because she had a magnetic charm of manner and her laugh was of the cheery kind until we were told that she under gaiety treasured a very sad memory and fidelity to one handsome, gallant youth in the days that pass, who having received her pledge of love departed to give up his life on the battle field for the cause he upheld and which she loves ardently yet. "Yes, you are full of flattery, you know you can not make me good looking, I may have had a certain amount of beauty in my youth; but the sad years since have caused too many wrinkles, too much gray hair and too much tan. No, I am an old woman now and with snaggled teeth. What can you do to improve the working effect of them, not to say looks? I do not care for the latter much. We assured her that we were sincere in our assertion that we could both improve her powers of mastication and contribute considerably towards enhancing her personal appearance. We twitted her that she was once a beauty and that the spell of her melodious voice had brought many a gay swain to her feet. Her eyes brightened and a slight bit of blush for a moment appeared on her wan cheek. When she answered reminiscently, Yes, the poor fellows, it was cruel in me to sing to them songs of love when the heart of the singer was cold and sad, but you know those who are the saddest often appear the gladdest. Those days are now so long gone but it is sweet to now and then turn back to them for a moment, but now I want my teeth fixed so I can chew some of my tough boarding house beef.

We rehabilitated her mouth with fillings, crowns and bridges, which she wittily pronounced as wonderful specimens of the skill of the jeweler and stone-cutter. "You do make me look better to be sure and if I can chew with all this jewelry in my mouth as well as it looks, I shall be more than satisfied and pleased; and if you will call around some evening I will attempt to sing to you, with what little voice I have left, one of those old time love songs with the arder left out." We accepted her invitation and made the call and had a delightful evening, her songs and reminiscences carrying us back more than a quarter of a century to an atmosphere that does not live now.

